# Analysis of soil fertility and toxic metal characteristics in open-pit mining areas in northern Shaanxi

**DOI:** 10.1038/s41598-024-52886-8

**Published:** 2024-01-27

**Authors:** Na Wang, Zhe Liu, Yingying Sun, Nan Lu, Yuhu Luo

**Affiliations:** 1 Institute of Land Engineering and Technology, Shaanxi Provincial Land Engineering Construction Group Co., Ltd., Xi’an, 710021 China; 2 Shaanxi Provincial Land Engineering Construction Group Co., Ltd., Xi’an, 710075 China; 3 Key Laboratory of Degraded and Unused Land Consolidation Engineering, Ministry of Natural Resources, Xi’an, 710021 China; 4 Shaanxi Engineering Research Center of Land Consolidation, Xi’an, 710021 China; 5 Land Engineering Technology Innovation Center, Ministry of Natural Resources, Xi’an, 710021 China

**Keywords:** Ecology, Ecology, Environmental sciences

## Abstract

The study specifically focused on the Hongliulin mining area, where a total of 40 soil samples were meticulously collected and analyzed from within a 1000 m radius extending from the tailings dam. The findings revealed that soil pH within the 0–1000 m range generally leaned towards the alkaline side. In terms of soil nutrient content, encompassing factors such as soil organic matter (SOM), total nitrogen (TN), total phosphorus (TP), total potassium (TK), alkali nitrogen (AK), available phosphorus (AP), and quick-acting potassium (AK), the variations fell within the following ranges: 2.23–13.58 g/kg, 0.12–0.73 g/kg, 0.18–1.15 g/kg, 9.54–35.82 g/kg, 2.89–6.76 mg/kg, 3.45–11.25 mg/kg, and 5.86–130.9 mg/kg. Collectively, these values indicate relatively low levels of soil nutrients. Within the 0–500 m range of soil samples, the average concentrations of Cd, Hg, Pb, and As were 0.778, 0.198, 24.87, and 17.92 mg/kg, respectively. These concentrations exceeded the established soil background values of Shaanxi Province and emerged as the primary pollutants in the study area. Within this same range, the mean values of eight toxic metals (Pi) were ranked in the following descending order: 1.726 (Hg), 1.400 (As), 1.129 (Cr), 1.109 (Pb), 0.623 (Zn), 0.536 (Cd), 0.309 (Cu), and 0.289 (Ni). With the exception of Hg, As, Cr, and Pb, which exhibited slight pollution, the other toxic metals were found to be within acceptable pollution limits for this sampling range, in line with the results obtained using the geo-accumulation index method. The average potential ecological risk index for the eight toxic metals in the study area stood at 185.0, indicating a moderate overall pollution level. When assessing individual elements, the proportions of ecological risk attributed to Hg, As, Pb, and Cd were 34.57%, 27.44%, 25.11%, and 23.11%, respectively. This suggests that the primary potential ecological risk elements in the study area are Hg and As, followed by Cd and Pb. Notably, toxic metals Hg and Pb, as well as As and Pb, exhibited significant positive correlations within the sampling area, suggesting a common source. An analysis of the relationship between soil physicochemical properties and toxic metals indicated that soil pH, SOM, TN, and TP were closely linked to toxic metal concentrations. The toxic metal elements in the research area's soil exhibit moderate variability (0.16 < CV < 0.36) to high variability (CV > 0.36). Within the range of 0–200 m, the CV values for Cd and Hg exceed 1, indicating a high level of variability. The coefficient of variation for SOM, TP, AP, AK and TK is relatively high with the of 2.93, 2.36, 2.36, 21.01, 7.54. The soil in the sampling area has undergone significant disturbances due to human activities, resulting in toxic metal pollution and nutrient deficiencies.

## Introduction

Due to the high toxicity, propensity for accumulation, and non-degradability of toxic metals, the increasing concentration of toxic metals in soil continues to pose a persistent threat to farmland quality and environmental safety^1,2^. In the natural environment, the sources of soil toxic metals can be broadly categorized into two main classes: natural sources and anthropogenic sources. Among these, the contribution of soil parent material weathering and geological components to soil toxic metal pollution is minimal. However, anthropogenic pollution, such as mining, chemical processing, urban traffic, and agricultural fertilization, significantly increases the soil burden of toxic metals and is considered the primary source of soil toxic metal pollution^3^. Previous studies have indicated that soil toxic metal contamination is closely associated with modern economic practices, project development, and the like. Non-degradable toxic metals enter the soil and are subsequently transferred to aquatic environments through processes such as rainfall and surface runoff, thereby further amplifying the ecological toxicity of these toxic metals^4,5^. Among all sources of toxic metal pollution, mining activities such as ore beneficiation, open-pit mining, tailings deposition, and transportation are the most significant contributors to environmental pollution due to the substantial release of toxic metal dust^6^. Existing studies have shown that metalloid arsenic (As) and toxic metals such as mercury (Hg), lead (Pb), cadmium (Cd), chromium (Cr), zinc (Zn), copper (Cu), and nickel (Ni) belong to highly toxic pollutants, posing a significant threat to the ecosystem in mining areas^7^. Furthermore, toxic metals can enter the human body through various pathways such as the skin and respiratory system, posing certain risks to human organs^8,9^ Prolonged inhalation of toxic metal dust can potentially lead to respiratory system diseases, birth defects, cardiovascular diseases, and central nervous system damage^10^. For instance, metals like Cu, Pb, and Cr can lead to diseases such as lung cancer, insomnia, memory impairment, gastrointestinal disorders, and, in extreme cases, even fatalities due to long-term exposure^3,11^. Many previous studies have assessed the health risks to human beings through non-carcinogenic and carcinogenic pathways of exposure to different toxic metals^12–14^. The substantial generation of toxic metal dust, stemming from activities such as overburden disposal, slag accumulation, and surface soil transportation in coal mining, is one of the primary causes of regional environmental pollution. Among these, open-pit mining imposes a more severe pollution burden on soil, water bodies, and the atmospheric environment. On the other hand, underground mining elevates the risk of ground subsidence and underground coal fires^15^. Coal dust contains various toxic metals including Fe, Zn, Mn, Cu, Pb, Cr, Ni, Sr, Zr, and As. These toxic metal-laden coal dust particles mix with surface soils in mining areas and can enter sub-surface soils, surface water, or groundwater through surface runoff^16,17^. The deterioration of water bodies, soil, and the ecological environment near mining areas directly introduces contaminants into the human body through the food chain, respiratory system, or skin contact, further exacerbating human health risks^18^. Many previous studies on soil pollution in coal mining areas have indicated that mining and its associated activities release toxic metals into nearby soils, subsequently contaminating farmland, surface water, and riverbank environments^19–22^. The Cd isotope compositions of contaminated soil near a Pb–Zn mine fell in a binary mixture between the ores and background signals, which suggested that soil Cd pollution was mainly attributed to the deposition of dust emitted by mining and refining activities^23^. The researches of Sun et al.^24^ also found that the surface soil was affected by sewage irrigation and wastewater discharge, and the Pb isotope tracing results demonstrated that sources of Pb were mainly coal (35.14%) in the some of the points. Research on soil toxic metal pollution in China has shown that the contamination levels of toxic metals such as Cd, Pb, and Hg have exceeded the concentration limits set by Chinese soil standards (Grade I and Grade II), indicating a high degree of pollution^25^. Currently, most of these studies combine GIS for spatial analysis of soil pollution in mining areas using various indicators^26^. However, there is limited research on the concentration and variation patterns of toxic metals in the soil around coal mining areas, as well as the study of the physicochemical properties of soil and their correlation with toxic metals.

The contiguous region encompassing the provinces of Shanxi, Shaanxi, and Inner Mongolia on the Loess Plateau is renowned for its extensive mining activities and soil erosion. Research has reported that approximately 77% of this area is affected by water erosion, with the highest erosion intensity reaching 22,000 t/km^2^/a^27^. In recent years, many scholars have applied geostatistical methods to study the spatial distribution of total nitrogen in the Loess Plateau region and its influencing factors^28^. However, there has not been a comprehensive analysis of the soil physicochemical properties, toxic metal pollution, and their interrelationships in this mining area. This study focuses on the Hongliulin coal mine in the contiguous region of Shanxi, Shaanxi, and Inner Mongolia, with the aim of investigating (1) the extent of soil pollution due to mining activities and tailings erosion, (2) identifying potential risks associated with various toxic elements and their correlations, and (3) assessing the interrelationships among soil physicochemical properties in the mining area.

## Materials and methods

### Study area

**Figure 1:**
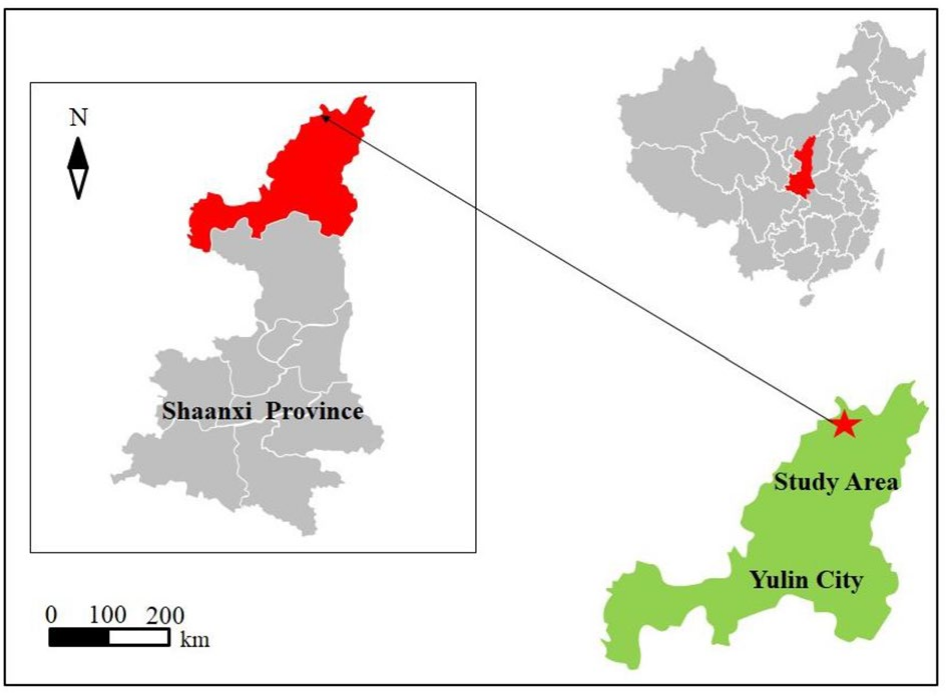
Location of study area.

The study area is located in the Hongliulin coalfield in Shenmu City, Shaanxi Province (Fig. 1). It is situated in the transitional zone between the hilly and gully region of northern Shaanxi's loess plateau and the Inner Mongolian grasslands. The area falls under a semi-arid continental monsoon climate, characterized by an elevation ranging from 987 to 1449 m. It predominantly experiences northwest winds, frequent sandstorms, a short frost-free period, extended daylight hours, and a high degree of heat accumulation. The region receives an annual average of 2860 h of sunshine, with a sunshine percentage of 65%. The total annual solar radiation is 141.9 kcal/cm^2^, and the biological radiation is 70.93 kcal/cm^2^, making it one of the areas in Shaanxi Province with abundant sunshine and strong radiation. The predominant soil types in the area are loess soil and sandy soil, characterized by loose soil structure, poor erosion resistance, and severe soil erosion. The primary land use types include farmland, grassland, forestland, industrial and mining land, and commercial land^27^.

### Sample collection and treatment

Samples were collected in August 2022, following the “Technical Specifications for Soil Environmental Monitoring” (HJ/T 166-2004) for site selection, sample collection, and sample processing. Soil samples were collected at distances of 0–100 m, 100–200 m, 200–500 m, and 500–1000 m around the tailings dam, totaling 40 samples. All samples were free from impurities such as plant roots and stones, placed in sample bags, air-dried naturally, and then sieved through 2 mm and 0.15 mm nylon sieves for the determination of soil physicochemical properties and toxic metal content.

### Sample analysis

#### Toxic metal analysis

The soil samples underwent digestion using a combined acid method, involving HCl-HNO_3_-HF-HClO_4_. Cu, Zn, and Ni content were quantified through flame atomic absorption spectroscopy, whereas Cd, Pb, and Cr were assessed using graphite furnace atomic absorption spectroscopy. Content was analyzed using cold atomic fluorescence spectroscopy. All reagents used in the entire analysis process were of analytical grade, and deionized water was used. Parallel duplicate samples were employed during the analysis, and the quality of measurements was controlled using national standard reference materials^29,30^. The study used standard reference material (GSS-8, GSS-10 and GSF-3) to assure the quality control. These materials are from the National Center for Standard Materials in China. The ratio of recoveries ranged between 90 and 110% for the elements throughout this study.

#### Soil physical and chemical properties analysis

Soil nutrient content analysis followed established national standard protocols. Soil pH was measured using a PHS-3B pH meter, following the guidelines outlined in “Determination of Soil pH” (NY/T 1121.2-2006). Soil organic matter content was determined using the potassium dichromate heating method, as described in “Part 6: Determination of Soil Organic Matter” within “Soil Detection” (NY/T 1121.6-2006). Soil total nitrogen was quantified using the Kjeldahl method, in accordance with “Soil Total Nitrogen Determination (Semimicro Kjeldahl Method)” (NY/T 53-1987). Soil alkali-hydrolyzable nitrogen was assessed using the alkali hydrolysis diffusion technique. For soil available phosphorus analysis, the 0.5 mol/L sodium bicarbonate extraction and molybdenum antimony anti-coloration method were employed, following “Part 7: Determination of Soil Available Phosphorus” within “Soil Detection” (NY/T 1121.7-2014). Soil total phosphorus was determined through the sodium hydroxide fusion-molybdenum antimony anti-coloration method, and soil total potassium was measured using the sodium hydroxide dissolution-flame photometer method. Both total phosphorus and total potassium analyses were conducted according to NY/T 88-1988. Soil available potassium was determined by extracting with a 1 mol/L neutral ammonium acetate solution and using the flame photometer method, following NY/T 889-2004 guidelines.

### Data analysis

Statistical analysis of the experimental data was conducted using Microsoft Excel 2010 and SPSS 19.0 software. Graphical representations were created using Origin Pro 9.0 and Sigmaplot 13.0 software.

### Receptor risk assessment methods

#### Single factor pollution index method

The single factor index is mainly used to evaluate the residual harm level of individual pollution elements in soil. Here, the Pi was calculated by the following Eq. (S1)

S1 $$P_{i} = \frac{{C_{i} }}{{S_{i} }}$$

where Pi is the pollution index of a certain pollutant, Ci is the measured toxic metal concentration, mg/kg, Si is the evaluation criterion of the pollutant, mg/kg. The evaluation standard for pollutants is based on the soil background value in Shaanxi Province.

#### Geo-accumulation index

The geo-accumulation pollution index method evaluates soil pollution by comparing the concentrations of toxic metals at sampling sites with the background values of toxic metals in the soil^31–33^. The formula for calculating Igeo is presented in Eq. (S2).

S2 $$I_{Geo} = Log_{2} \left( {\frac{{C_{i} }}{{kB_{i} }}} \right)$$

In this context, C_i_ represents the recorded concentration of toxic metal i (mg/kg); B_i_ signifies the natural background value of toxic metals and metalloids in Shaanxi province, with a conversion coefficient denoted as k, which equals 1.5. The classification of toxic metals and metalloids into seven levels is based on the magnitude of the values, as outlined in Table 1.

**Table 1:** Geochemical index classification.

*I*_*Geo*_	Classification	*I*_*Geo*_	Classification
*I*_*Geo*_ < 0	Pure	2 ≤ *I*_*Geo*_ < 3	Moderately to heavily contaminated
0 ≤ *I*_*Geo*_ < 1	Uncontaminated to mildly polluted	3 ≤ *I*_*Geo*_ < 4	Severely polluted
1 ≤ *I*_*Geo*_ < 2	Moderately polluted	4 ≤ *I*_*Geo*_ < 5	Strongly to extremely polluted
*I*_*Geo*_ ≥ 6	Incredibly polluted		

*I*_*Geo*_ stands for the Geoaccumulation Pollution Index.

#### Potential ecological risk analysis

The Potential Ecological Hazard Index method, originally introduced by the Swedish scientist Hakansson^34^, is a quantitative approach for assessing the potential ecological risk posed by specific pollutants. It achieves this by incorporating toxicological, environmental, and ecological factors into an index-based grading system. This method is valued for its comparability and equivalence. When gauging the ecological risk associated with toxic metals, it has the capacity to not only reflect the individual impact of distinct toxic metal elements in the soil but also account for their collective effects. Consequently, it enables a more comprehensive evaluation of the pollution status and potential risks posed by toxic metals in the soil environment^35^. The formula for calculating the Potential Ecological Hazard Index is as follows:

S3 $$C_{f}^{i} = \frac{{c_{s}^{i} }}{{c_{n}^{i} }}$$

S4 $$E_{r}^{i} = T_{r}^{i} \times C_{f}^{i}$$

S5 $$RI = \sum\limits_{i = 1}^{n} {E_{r}^{i} }$$

In the formula, ${\text{C}}_{{\text{s}}}^{{\text{i}}}$ represents the measured content of element i, ${\text{C}}_{{\text{n}}}^{{\text{i}}}$ represents the reference value for element i (the background values for soil environmental elements in Shaanxi Province), ${\text{E}}_{{\text{r}}}^{{\text{i}}}$ is the potential ecological hazard coefficient for toxic metal element i, ${\text{T}}_{{\text{r}}}^{{\text{i}}}$ is the toxicity response coefficient for toxic metal i, and RI is the potential ecological risk index.

According to the standardized toxic metal toxicity coefficients established by Hakansson, as shown in Table 2, the evaluation criteria for the ecological hazard level of the potential ecological hazard coefficient ${\text{E}}_{{\text{r}}}^{{\text{i}}}$ and hazard index RI are presented in Table 3.

**Table 2:** Environmental baseline levels and toxicological response parameters of toxic metals in soil^36,37^.

Element	Cd	Cr	Hg	As	Pb	Cu	Zn	Ni
$c_{n}^{i}$ (mg/kg)	0.76	62.5	0.063	11.1	21.4	21.4	69.4	28.8
$T_{r}^{i}$	30	2	40	10	5	5	1	5

$c_{n}^{i}$ stands for the reference value for element *i*, $T_{r}^{i}$ stands for the toxicity response coefficient for toxic metal *i*.

**Table 3:** Classification of potential ecological risk coefficient (${\text{E}}_{{\text{r}}}^{{\text{i}}}$) and risk index (RI).

Ecological risk	Low risk (A)	Moderate risk (B)	Considerable risk (C)	High risk (D)	Significantly high risk (E)
$E_{r}^{i}$	< 40	40–80	80–160	160–320	> 320
RI	< 150	150–300	300–600	≥ 600	–

$E_{r}^{i}$ stands for the potential ecological hazard coefficient, *RI* stands for the Potential Ecological Risk Index.

## Results and discussion

### Analysis of changes in soil chemical properties

The soil pH, SOM, TN, AHN, TP, TK, and AP were analyzed in the study area, and statistical analyses were conducted across four different zones, resulting in a Soil Chemical Properties Statistical Table (Table 4).

**Table 4:** Contents of soil nutrient elements in mine tailings.

Distance/m	pH		SOM		TN		AHN	
	Value	CV	Value g/kg	CV	Value g/kg	CV	Value mg/kg	CV
0–1000	7.46–9.09	0.40	2.23–13.58	2.93	0.12–0.73	0.16	2.89–6.76	1.15
	8.53		7.74		0.44		4.72	
0–100	8.08–8.48	0.20	8.22–13.58	2.32	0.12–0.66	0.24	3.65–6.76	1.32
	8.28		10.40		0.40		4.92	
100–200	8.47–9.04	0.28	6.25–11.50	2.09	0.33–0.58	0.11	2.89–5.58	1.18
	8.73		8.91		0.46		4.25	
200–500	7.46–9.09	0.60	2.23–11.22	3.42	0.22–0.73	0.20	0.24–6.66	1.35
	8.58		7.06		0.43		4.76	
500–1000	8.28–8.59	0.12	4.65–6.26	0.67	0.28–0.57	0.13	3.98–5.88	0.85
	8.47		5.39		0.44		4.97	

Distance/m	TP		AP		TK		AK	
	Value g/kg	CV	Value mg/kg	CV	Value g/kg	CV	Value mg/kg	CV
0–1000	0.18– 1.15	0.31	3.45–11.25	2.36	9.54–35.82	7.54	45.86–130.85	21.07
	0.54		6.91		22.43		94.16	
0–100	0.28–0.33	0.02	3.45–9.55	2.73	19.85–31.05	4.59	85.69–99.68	6.50
	0.31		6.70		25.78		95.36	
100–200	0.34–0.64	0.11	3.68–11.25	3.13	16.98–30.02	6.37	98.45–125.35	11.34
	0.47		3.79		23.63		107.19	
200–500	0.18–1.02	0.36	3.68–9.88	2.16	9.54–35.82	8.86	67.89–130.85	23.32
	0.60		6.87		18.00		99.01	
500–1000	0.22–1.15	0.39	6.89–10.12	1.25	15.65–33.94	7.48	45.86–98.99	21.21
	0.72		8.28		24.77		73.37	

*CV* stands for the coefficient of variation.

The magnitude of soil pH not only influences vegetation growth but is also a crucial factor in soil fertility, especially concerning nutrient release and soil microbial activity. It has been reported that pH is an important factor affecting the mobility and solubility of soil toxic metal cations^37,38^. In the study area, the soil tends to be slightly alkaline, with an average pH of 8.53 and a range of 7.46–9.09. The coefficient of variation is 0.398. This condition is not conducive to the transfer of toxic metals from the soil to plants^39^. Within the 0–100 m range, the soil pH has an average of 8.28, which is the lowest in the entire area. This is likely due to the mining activities that have introduced acidic substances into the soil, causing a decrease in pH. In the 100–200 m region, the soil pH measures 8.73, the highest across the entire area. Within the 200–1000 m zone, the pH gradually decreases. This can be attributed, on one hand, to the natural decomposition of organic matter increasing soil acidity and, on the other hand, to the nitrogen-fixing activity of vegetation leading to higher soil acidity.

SOM is one of the crucial components of soil. It not only enhances the effectiveness of soil nutrients but also promotes the formation of soil aggregates, thus improving soil physical properties. It is a primary assessment indicator for ecological restoration^40,41^. In mining soils, the accumulation of organic matter and organic carbon was previously considered a key factor in activating soil biological processes^42^. In the study area, the SOM content is generally low, with an average of only 7.74 g/kg. According to the nutrient classification standards from the second national soil survey, this falls within the fifth nutrient level, with a range of 2.23–13.58 g/kg. However, the coefficient of variation is relatively high at 2.93. Within the 0–100 m region, the SOM content is highest, with an average of 10.40 g/kg and a range of 8.22–13.58 g/kg. In this area, the soil appears dark gray due to the presence of coal dust, as coal is primarily composed of carbon. This results in significantly higher organic matter content compared to other sampling points. As the sampling distance increases, the SOM content gradually decreases.

TN is a necessary nutrient element for plant growth and development. It helps maintain soil ecological nutrient balance, stimulates the growth of underground plant roots, improves soil quality, and is one of the important indicators of soil fertility. In the study area, soil TN content is generally low, with an average of 0.44 g/kg, falling within the sixth nutrient level, with a range of 0.12–0.73 g/kg. Across the entire sampling area, soil TN exhibits minimal fluctuations with distance. The soil available nitrogen content in the sampling area has an average of 4.72 mg/kg, with a range of 2.89–6.76 mg/kg, and a relatively high coefficient of variation at 1.15. As sampling points increase in distance, available nitrogen shows relatively stable fluctuations. In general, nitrogen and organic matter content in mining area soils tend to be low, which limits the establishment of vegetation and sustainable productivity. This study's findings are consistent with these trends^43,44^.

The average soil TP content in the study area is 0.54 g/kg, indicating a fourth nutrient level, with a range between 0.18 and 1.15. The coefficient of variation is 0.305. With increasing sampling distance, the soil TP content exhibits a rising trend, reaching its peak within the range of 500–1000 m, with an average content of 0.72 g/kg. Soil AP is a sensitive indicator of plant-absorbable phosphate content in soil and an important reflection of soil conditions. The mean soil AP content in the study area is 6.91 mg/kg, corresponding to a fourth nutrient level, with a range between 3.45 and 11.25 mg/kg. The coefficient of variation is 2.36. AP displays significant fluctuations with distance: within the 0–500 m, it initially decreases and then increases, while within the 500–1000 m, it reaches its highest point at 8.28 mg/kg.

The soil in the study area is relatively rich in TK, with an average content of 22.43 g/kg, reaching a second nutrient level, ranging from 9.54 to 35.82 g/kg. With increasing sampling distance, the TK content shows a trend of first decreasing and then increasing. Within the 200–500 m sampling interval, the average TK content is at its lowest, at 18.00 g/kg, with a range of 9.54–35.82 g/kg. The coefficient of variation is exceptionally high at 8.86. Soil AK, which is more readily absorbed and utilized by plants, is an important indicator of potassium levels. In the study area, the average soil AK content is 94.16 mg/kg, indicating a fourth nutrient level (moderate), with a range between 5.86–130.85 mg/kg. The coefficient of variation is extremely high at 21.08. Across the entire sampling area, AK exhibits a trend of first increasing and then decreasing. Within the 100–200 m sampling interval, the average AK content reaches its highest point at 107.2 mg/kg, with a range of 98.45–125.4 mg/kg. Within the 500–1000 m sampling interval, the average AK content reaches its lowest point at 73.37 mg/kg, with a range of 45.86–98.99 mg/kg.

### Characteristics of toxic metal pollution

**Figure 2:**
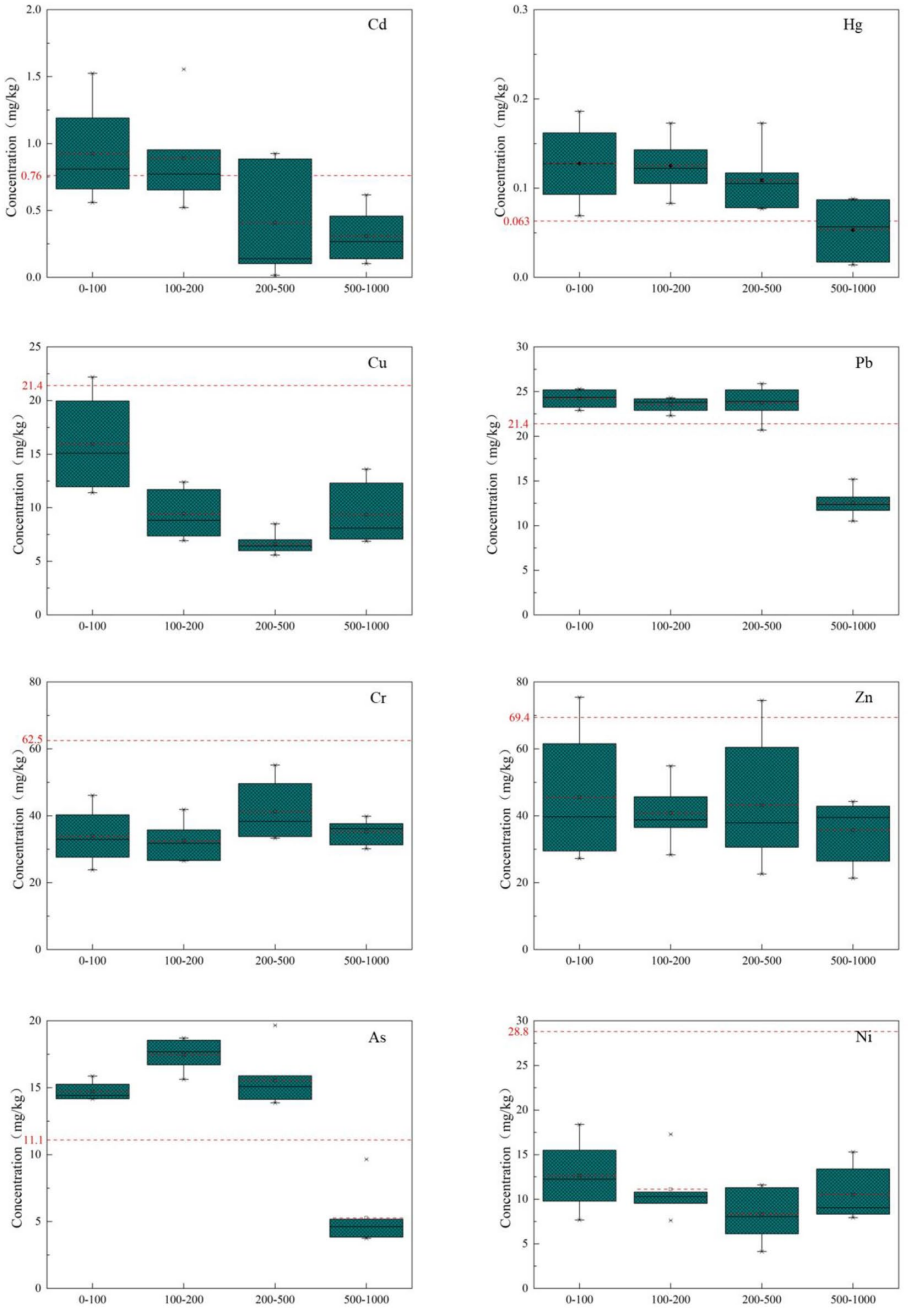
Toxic metal concentration in the soil of the study area.

Within the 0–1000 m range of the study area, soil pH values are greater than 7.5, indicating that the predominant soil type in the study area is alkaline (Table 4). Soil toxic metal content, as shown in Fig. 2, was assessed against the screening values for agricultural land soil pollution risk (GB15618-2018, pH > 7.5). In the 0–500 m range, the toxic metal primarily exceeding the standard is Cd, with average contents of 0.926 mg/kg (0–100 m) and 0.891 mg/kg (100–200 m), both surpassing the screening values for agricultural land soil pollution risk, with excess rates of 70% and 60%, respectively. The average toxic metal content in other soil samples is lower than the screening values for agricultural land soil pollution risk. In the 0-500 m range, the average Cd, Hg, Pb, and As contents all exceed the soil background values of Shaanxi Province. Specifically, in the 0–100 m range, the average Cd, Hg, Pb, and As contents are 1.23, 2.02, 1.13, and 1.33 times higher than the soil background values of Shaanxi Province, respectively. In the 100–200 m range, the average Cd, Hg, Pb, and As contents are 1.17, 1.99, 1.11, and 1.57 times higher than the soil background values of Shaanxi Province, respectively. In the 200–500 m range, the average Hg, Pb, and As contents are 1.73, 1.12, and 1.40 times higher than the soil background values of Shaanxi Province, respectively. All of these values exceeded their respective standard levels, indicating a significant accumulation of toxic metals in the soil^7,45^.

There is a significant variation in soil toxic metal content among sampling points at different distances. Similar results were also observed in groundwater sampling by Qiao et al.^46^. According to reports, the average concentration of lead (Pb) in farmland soils affected by coal mining reaches as high as 433 mg kg^−1^^47^. This is primarily attributed to the presence of Pb in coal-associated minerals and sulfides, such as PbS, PbSe^48^, pyrite, as well as aluminum silicates and carbonates^49^. Additionally, Mishra et al.^50^ and Cao et al.^51^ have pointed out in their research that As is a primary toxic metal pollutant during coal mining and utilization, which is consistent with the findings of this study. Overall, the average concentrations of toxic metals Cd, Hg, Pb, and As tend to decrease with increasing distance. The remaining toxic metals, Cu, Cr, Zn, and Ni, as well as their average concentrations, are all below the background values for soil in Shaanxi Province, indicating a relatively lower level of regional ecological environmental pollution.

The coefficient of variation (CV) is a normalized measure of the degree of dispersion in a probability distribution. Generally, a higher degree of dispersion leads to a larger coefficient of variation, indicating a greater influence of human factors on the distribution. Factors such as the application of fertilizers and pesticides, industrial activities, transportation, and mining pollution can all result in surface enrichment of toxic metal in soil. A smaller coefficient of variation suggests that natural sources are the primary contributors to these toxic metals^52^.

The research area is densely populated with coal mining activities, significant vehicular traffic, and relatively concentrated human production and living, which are likely the primary sources of pollution. Based on relevant studies and the classification of the coefficient of variation (CV) size, the toxic metal elements in the research area's soil exhibit moderate variability (0.16 < CV < 0.36) to high variability (CV > 0.36) (Table 5). Within the range of 0–200 m, the CV values for Cd and Hg exceed 1, indicating a high level of variability. The toxic metal elements in the research area exhibit a high degree of variation, significant spatial dispersion, and are strongly influenced by human factors. This indicates the uneven distribution of toxic metals in coal mine. Comparable toxic metal and CV values were also reported in nine coal mines in Shanxi Province^53^.

**Table 5:** Variation coefficient and exceeding standard of toxic metals in the soil of the study area.

	Distance (m)	Cd	Hg	Pb	As
CV	0–100	1.11	1.39	0.61	0.63
	100–200	1.23	1.15	0.55	0.47
	200–500	0.98	0.68	0.31	0.36
	500–1000	0.84	0.55	0.28	0.21
Excessive rates %	0–100	70	100	100	100
	100–200	60	100	100	100
	200–500	25	100	100	100
	500–1000	0	35	0	0

*CV* stands for the coefficient of variation. The coefficient of variation and exceedance rate of toxic metal content are dimensionless, and the exceedance rate is calculated by comparing the soil background values in Shaanxi Province, expressed as a percentage.

### Soil toxic metal pollution assessment

**Figure 3:**
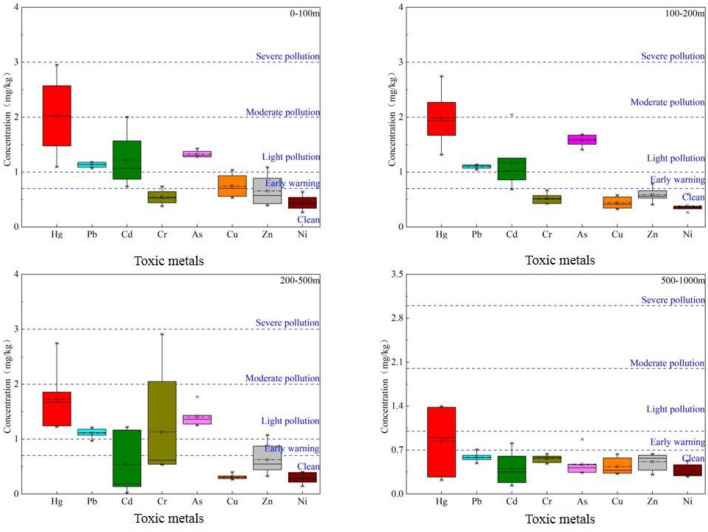
Single factor pollution index.

The single-factor pollution assessment in this study (Fig. 3) indicates that the Pollution index (Pi) for Hg, Pb, Cd, Cr, As, Cu, Zn, and Ni in the research area range from 0.222–2.952, 0.491–1.210, 0.021–2.046, 0.381–2.912, 0.338–1.771, 0.261–1.037, 0.307–1.086, and 0.143–0.639, respectively. Comparing the Pi values for different distances, within the 0–100 m range, the mean Pi values for these 8 toxic metals, in descending order, are 2.024 (Hg), 1.326 (As), 1.218 (Cd), 1.132 (Pb), 0.745 (Cu), 0.656 (Zn), 0.543 (Cr), and 0.439 (Ni). This suggests that, except for Hg, which is moderately polluted, the remaining toxic metals are at levels of mild pollution or lower. Within the 100–200 m range, the mean Pi values for these 8 toxic metals, in descending order, are 1.987 (Hg), 1.573 (As), 1.172 (Cd), 1.098 (Pb), 0.588 (Zn), 0.520 (Cr), 0.441 (Cu), and 0.386 (Ni). Except for Hg, As, Pb, and Cd, which are mildly polluted, the other toxic metals show no pollution within this sampling range. Within the 200–500 m range, the mean Pi values for these 8 toxic metals, in descending order, are 1.726 (Hg), 1.400 (As), 1.129 (Cr), 1.109 (Pb), 0.623 (Zn), 0.536 (Cd), 0.309 (Cu), and 0.289 (Ni). Apart from Hg, As, Cr, and Pb, which are mildly polluted, the other toxic metals show no pollution within this sampling range. Within the 500–1000 m range, the mean Pi values for these 8 toxic metals are all below 1, indicating no pollution for these toxic metals within this sampling range.

### Geo-accumulation evaluation index

The Geo-accumulation Index method, proposed by Muller^54^, is a quantitative assessment approach used to study the extent of toxic metal pollution in sediments and other substances. This method can effectively depict the distribution characteristics of toxic metal pollution in soils while taking into account factors such as anthropogenic pollution, geochemical background values, and natural diagenesis processes^55,56^. In recent years, it has been widely adopted by scholars worldwide for assessing soil toxic metal pollution. Compared to other single-factor pollution assessment methods, the advantage of the Geo-accumulation Index lies in its more precise evaluation scale^52^.

**Figure 4:**
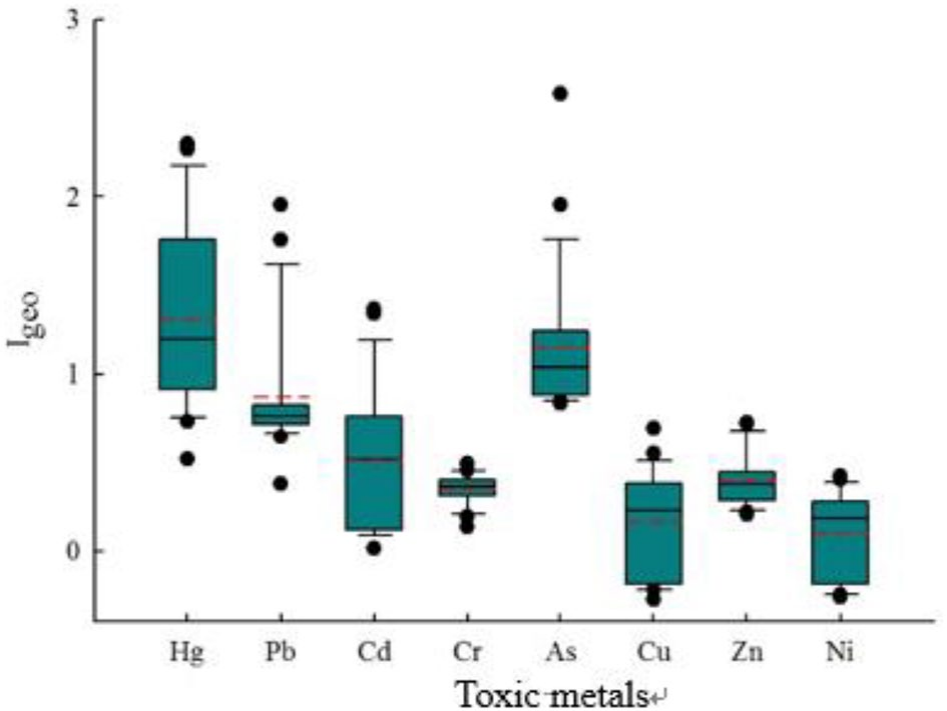
The boxplot of geo-accumulation indexes for toxic metals in soil.

Based on the soil element background values in Shaanxi Province, the Geo-accumulation Index (Igeo) of soil toxic metal elements in the research area (Fig. 4) was calculated. The range of Geo-accumulation Index values for the study area is as follows: Hg (0.52–2.30), Pb (0.38–1.95), Cd (0.01–1.36), Cr (0.17–0.49), As (0.83–2.58), Cu (− 0.28 to 0.69), Zn (0.20–0.72), and Ni (− 0.26 to − 0.43). With the exception of some sampling points for Cu and Ni where the Geo-accumulation index is less than 0, the Igeo values for the other toxic metals are greater than 0. This indicates that the soil at these sampling points has been polluted by Hg, Pb, Cd, Cr, As, and Zn. Additionally, for Pb and Cd, there are soil samples with Igeo values exceeding 1, suggesting that localized pollution for these four toxic metals has reached a moderate level. Furthermore, for Hg and As, there are soil samples with Igeo values exceeding 2, indicating a severe level of localized pollution for Hg and As. Overall, the order of soil toxic metal pollution levels in the study area, from highest to lowest, is as follows: Hg > As > Pb > Cd > Zn > Cr > Cu > Ni, which is generally consistent with the results obtained from the single-factor pollution assessment.

### Potential ecological risk assessment

The potential ecological risk assessment index is employed for a quantitative evaluation of the ecological risk posed by toxic metals in soils and sediments, as established by Wu et al.^57^. The Potential Ecological Risk Index (RI) for toxic metals, as illustrated in Table 6, has an average score of 185.00. This suggests that the overall ecological risk associated with soil toxic metal pollution in the study region falls within the moderate range. The average RI coefficients for individual elements are as follows: Hg (63.95) > As (50.76) > Cd (46.45) > Pb (42.75) > Cu (2.27) > Ni (1.80) > Cr (1.46) > Zn (0.56). Comparing the values in Table 6, Cu, Ni, Cr, and Zn are generally associated with mild potential ecological risks, while Hg, As, Pb, and Cd pose a moderate potential ecological risk. The percentage contributions to ecological risk for Hg, As, Pb, and Cd elements are 34.57%, 27.44%, 25.11%, and 23.11%, respectively. This indicates that the primary potential ecological risk elements in the study area are Hg and As, followed by Cd and Pb. The higher ecological risk associated with Hg and Cd in the soil aligns with previous research on soil^58,59^, sediments^60^, and lakes^61^.

**Table 6:** Potential ecological risk index statistics.

Item	Elements	Average	Min	Max	Std	CV
${\text{E}}_{{\text{r}}}^{{\text{i}}}$	Hg	63.95	8.90	118.1	28.16	0.45
	Pb	42.75	24.04	75.96	15.85	0.38
	Cd	46.45	4.03	61.38	15.50	0.74
	Cr	1.46	0.76	5.82	1.15	0.81
	As	50.76	13.20	104.1	23.01	0.46
	Cu	2.27	1.30	5.19	0.96	0.43
	Zn	0.56	0.31	0.87	0.15	0.27
	Ni	1.80	0.72	3.19	0.59	0.34
RI		185.0	76.67	290.3	52.46	0.29

*Std* stands for standard deviation.

### Correlation analysis of soil toxic metals and physicochemical properties

**Figure 5:**
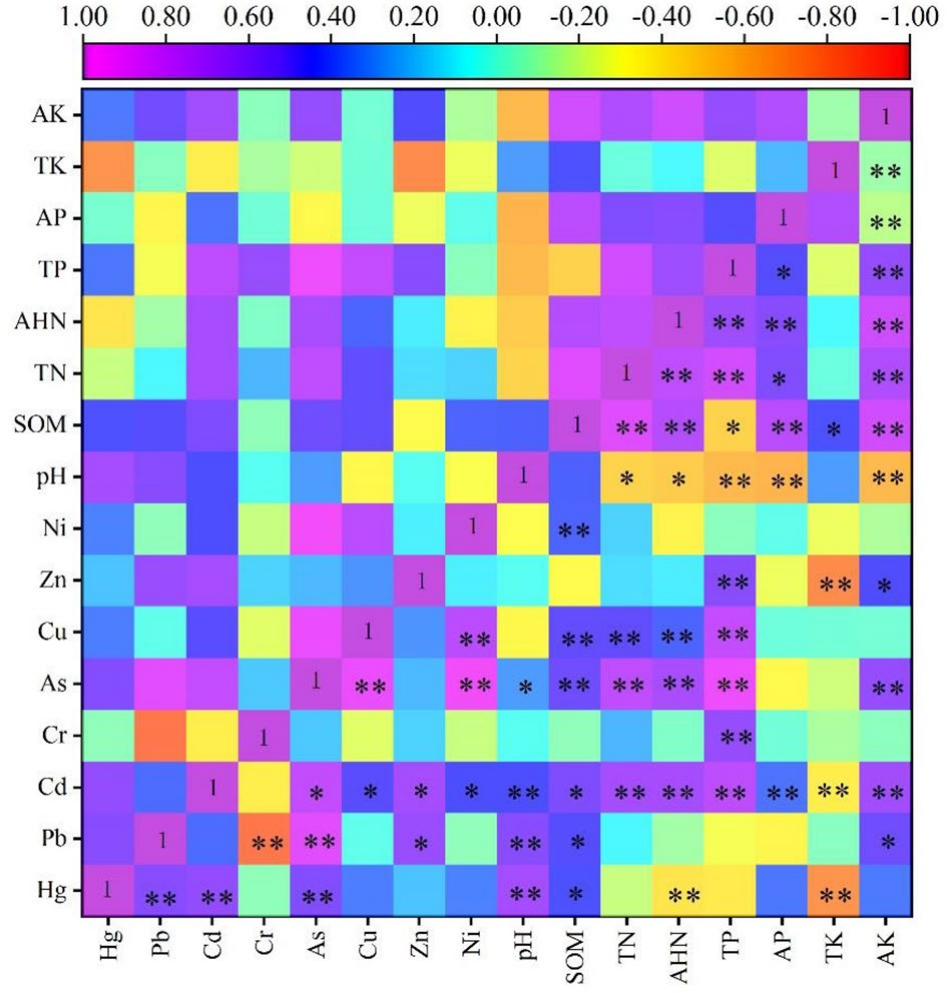
Heat map of correlation analysis.

Toxic metals and nutrient parameters serve as crucial indicators for assessing soils and rocks. Previous research has identified positive associations between soil nutrient characteristics, pH levels, and toxic metal concentrations in the soil^62,63^. Notably, correlation coefficients exceeding 0.5 were observed for toxic metals Hg and Pb, as well as Hg and As, indicating a strong positive correlation (*P* < 0.01) (Fig. 5). This suggests a pronounced interrelationship among these three elements during the migration of environmental media, implying a common source for these substances. Furthermore, a significant negative correlation was detected between toxic metal Pb and Cr, with a correlation coefficient of -0.816, while positive correlations were observed with As and Zn, showing correlation coefficients of 0.880 and 0.630, respectively. In the case of Cd, it exhibited positive correlations with As, Cu, Zn, and Ni, with relatively high correlation coefficients for Cd–As and Cd-Zn at 0.772 and 0.671, respectively. Similarly, As demonstrated substantial positive correlations with Cu and Ni, exhibiting correlation coefficients of 0.911 and 0.981, respectively, indicating a common source among these elements^64^.

There is also a varying degree of correlation between soil physicochemical properties and toxic metals. Among them, pH, SOM, TP, and toxic metals in the soil are closely related. For the Cd, it is negatively correlated with TK but significantly positively correlated with pH, TN, AHN, TP, AP, and AK, with the highest correlation coefficient observed with TP at 0.760. The activity of toxic metals Cu and As is largely influenced by SOM, TN, AHN, and TP. SOM shows positive correlations with most toxic metals except for Cr and Zn. SOM can affect toxic metal content through various pathways and, with increasing content, alter soil colloid structure, enhancing adsorption. Analyzing the soil physicochemical properties at sampling points, it is found that pH is unrelated to SOM and TK but has a negative correlation with TN, AHN, AP, TP, and AK. SOM has a strong correlation with TN, AHN, AP, and AK, with correlation coefficients exceeding 0.7. TN and AHN are significantly correlated with TP, AP, and AK, except for TK. This study aligns with similar research by Liu et al.^65^.

## Conclusion

The study investigated the concentrations of toxic metals, potential ecological risks, and the physicochemical properties of soils at various sampling points in the Hongliulin coal mine in northern Shaanxi. The soils in the study area are generally alkaline, with relatively low nutrient content in terms of TN, SOM, and TK. Additionally, nutrient content tends to increase with distance.

Within the 0–1000 m sampling range, Cd, Hg, Pb, and As are the main pollutants, with their average concentrations exceeding the soil background values of Shaanxi Province. Single-factor pollution index indicate that Hg, As, Cr, and Pb are mainly concentrated within the 0–500 m sampling points, a result corroborated by geo-accumulation index evaluations. The potential ecological risk assessment reveals that Hg and As are the primary ecological risk elements in the study area, followed by Cd and Pb. Hg and Pb, along with As and Cd, exhibit significant correlation coefficients, indicating a high degree of correlation and a common source. Soil organic matter (SOM), nitrogen (N), and phosphorus (P) have a substantial influence on the toxic metals in the soil.

## Data Availability

The primary findings and contributions presented in this study are detailed within the article and supplementary material. For additional queries or information, please contact the corresponding author.
